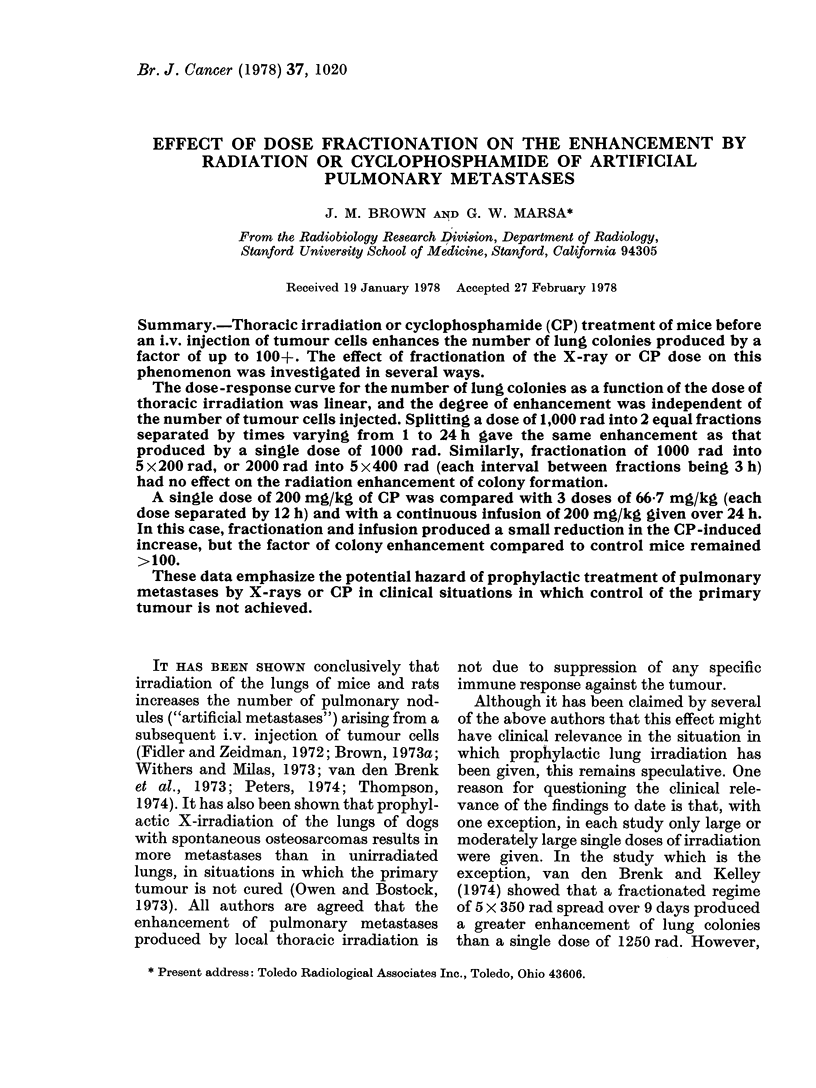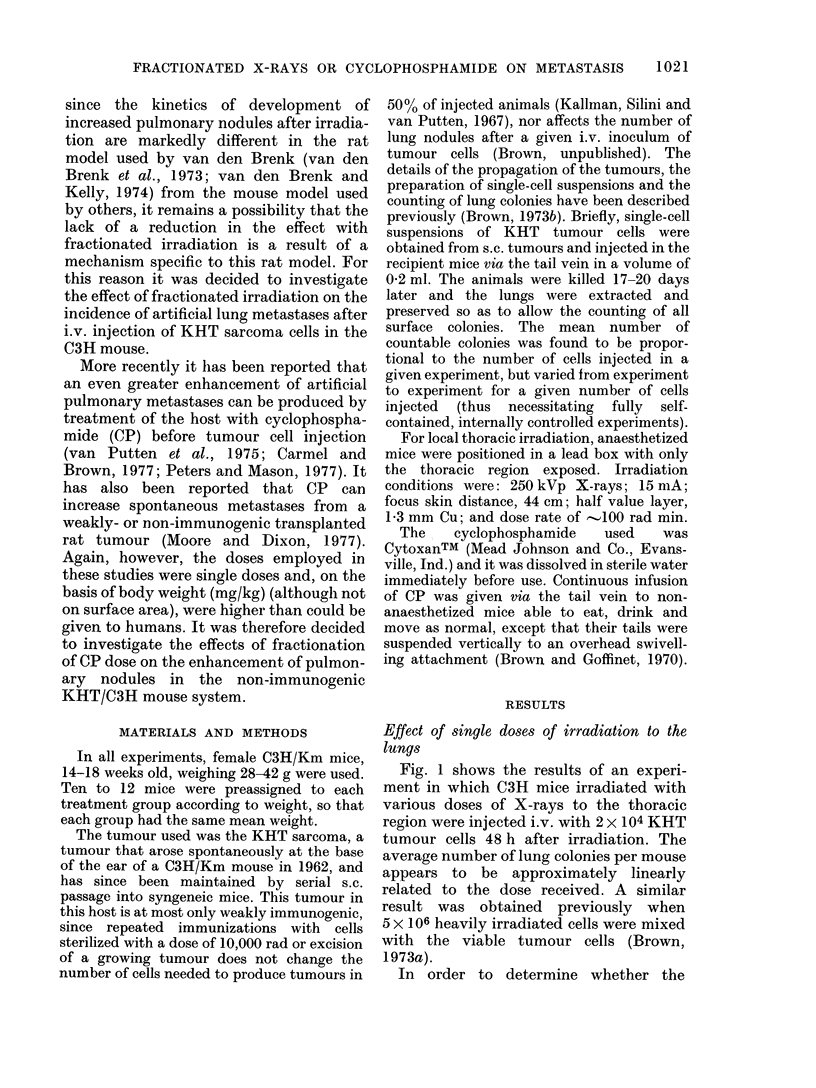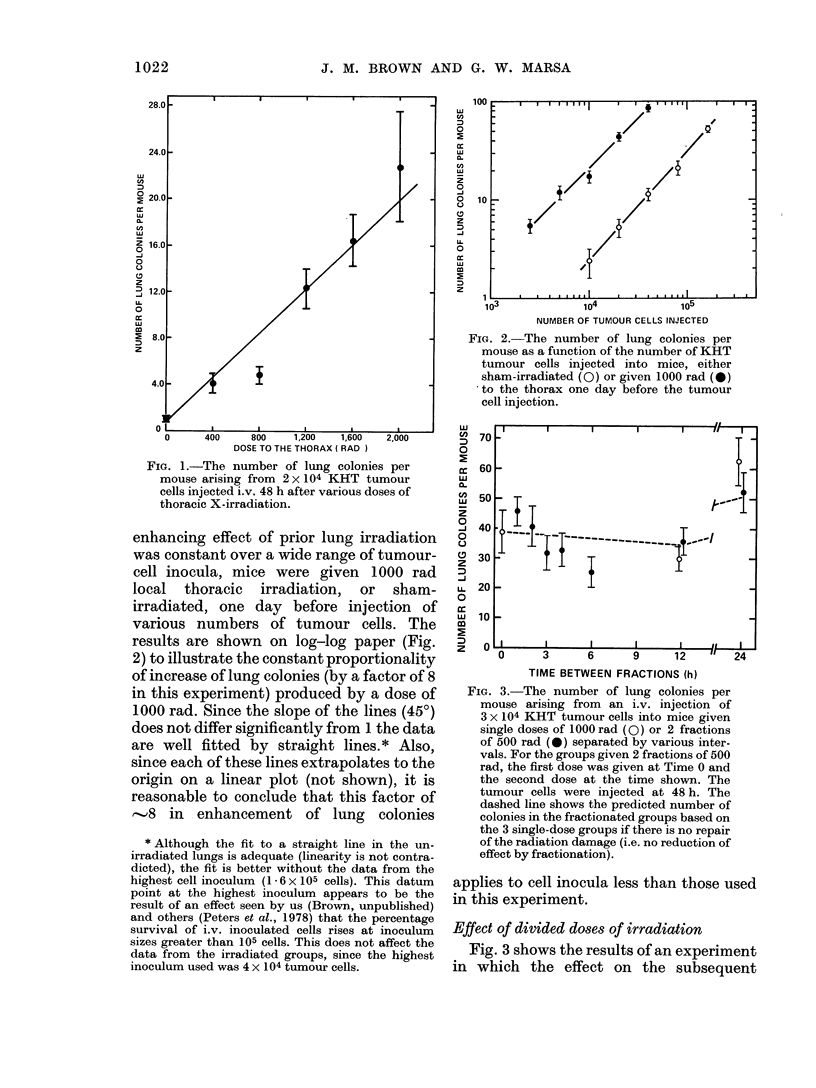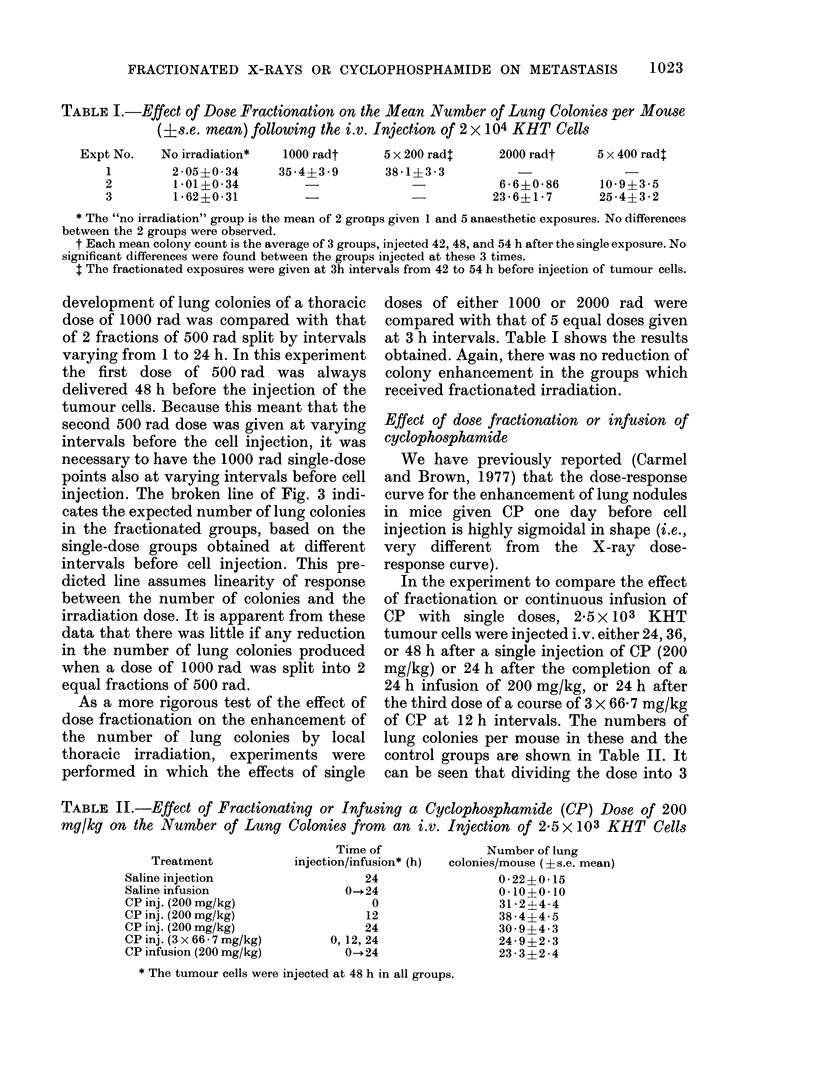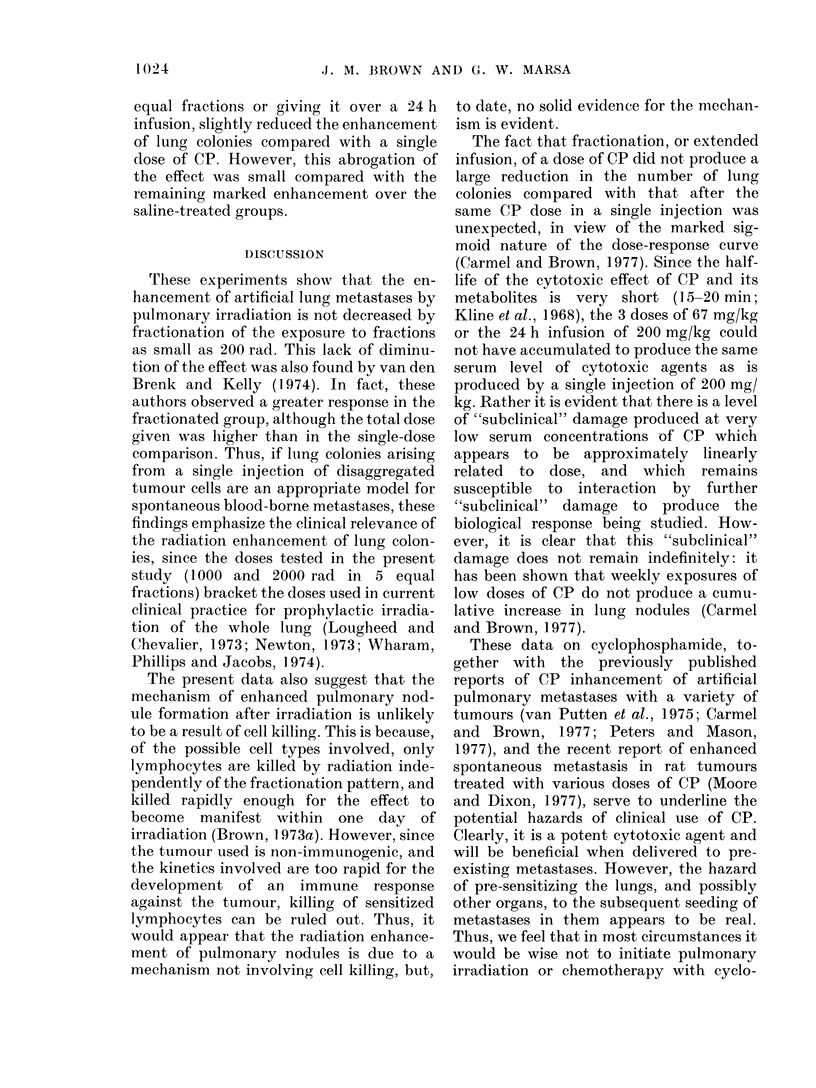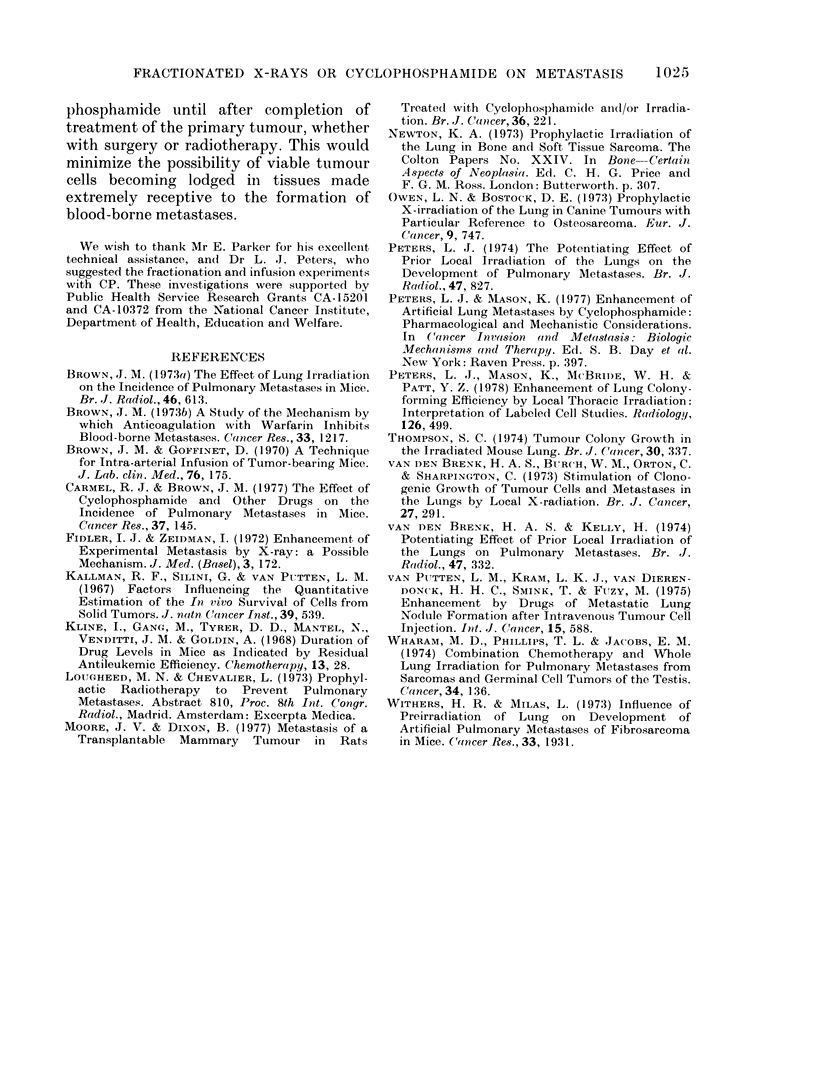# Effect of Dose Fractionation on the Enhancement by Radiation or Cyclophosphamide of Artificial Pulmonary Metastases

**DOI:** 10.1038/bjc.1978.148

**Published:** 1978-06

**Authors:** J. M. Brown, G. W. Marsa

## Abstract

Thoracic irradiation or cyclophosphamide (CP) treatment of mice before an i.v. injection of tumour cells enhances the number of lung colonies produced by a factor of up to 100+. The effect of fractionation of the X-ray or CP dose on this phenomenon was investigated in several ways.

The dose-response curve for the number of lung colonies as a function of the dose of thoracic irradiation was linear, and the degree of enhancement was independent of the number of tumour cells injected. Splitting a dose of 1,000 rad into 2 equal fractions separated by times varying from 1 to 24 h gave the same enhancement as that produced by a single dose of 1000 rad. Similarly, fractionation of 1000 rad into 5 × 200 rad, or 2000 rad into 5 × 400 rad (each interval between fractions being 3 h) had no effect on the radiation enhancement of colony formation.

A single dose of 200 mg/kg of CP was compared with 3 doses of 66·7 mg/kg (each dose separated by 12 h) and with a continuous infusion of 200 mg/kg given over 24 h. In this case, fractionation and infusion produced a small reduction in the CP-induced increase, but the factor of colony enhancement compared to control mice remained >100.

These data emphasize the potential hazard of prophylactic treatment of pulmonary metastases by X-rays or CP in clinical situations in which control of the primary tumour is not achieved.


*Br. J. Cancer* (1978) **37**, 1020

### EFFECT OF DOSE FRACTIONATION ON THE ENHANCEMENT BY RADIATION OR CYCLOPHOSPHAMIDE OF ARTIFICIAL PULMONARY METASTASES

J. M. BROWN AND G. W. MARSA*

*From the Radiobiology Research Division, Department of Radiology, Stanford University School of Medicine, Stanford, California* 94305

Received 19 January 1978 Accepted 27 February 1978

**Summary.—Thoracic irradiation or cyclophosphamide (CP) treatment of mice before an i.v. injection of tumour cells enhances the number of lung colonies produced by a factor of up to 100+. The effect of fractionation of the X-ray or CP dose on this phenomenon was investigated in several ways.**

**The dose-response curve for the number of lung colonies as a function of the dose of thoracic irradiation was linear, and the degree of enhancement was independent of the number of tumour cells injected. Splitting a dose of 1,000 rad into 2 equal fractions separated by times varying from 1 to 24 h gave the same enhancement as that produced by a single dose of 1000 rad. Similarly, fractionation of 1000 rad into 5×200 rad, or 2000 rad into 5×400 rad (each interval between fractions being 3 h) had no effect on the radiation enhancement of colony formation.**

**A single dose of 200 mg/kg of CP was compared with 3 doses of 66·7 mg/kg (each dose separated by 12 h) and with a continuous infusion of 200 mg/kg given over 24 h. In this case, fractionation and infusion produced a small reduction in the CP-induced increase, but the factor of colony enhancement compared to control mice remained >100.**

**These data emphasize the potential hazard of prophylactic treatment of pulmonary metastases by X-rays or CP in clinical situations in which control of the primary tumour is not achieved.**

IT HAS BEEN SHOWN conclusively that irradiation of the lungs of mice and rats increases the number of pulmonary nodules ("artificial metastases") arising from a subsequent i.v. injection of tumour cells (Fidler and Zeidman, 1972; Brown, 1973*a*; Withers and Milas, 1973; van den Brenk *et al.*, 1973; Peters, 1974; Thompson, 1974). It has also been shown that prophylactic X-irradiation of the lungs of dogs with spontaneous osteosarcomas results in more metastases than in unirradiated lungs, in situations in which the primary tumour is not cured (Owen and Bostock, 1973). All authors are agreed that the enhancement of pulmonary metastases produced by local thoracic irradiation is not due to suppression of any specific immune response against the tumour.

Although it has been claimed by several of the above authors that this effect might have clinical relevance in the situation in which prophylactic lung irradiation has been given, this remains speculative. One reason for questioning the clinical relevance of the findings to date is that, with one exception, in each study only large or moderately large single doses of irradiation were given. In the study which is the exception, van den Brenk and Kelley (1974) showed that a fractionated regime of 5×350 rad spread over 9 days produced a greater enhancement of lung colonies than a single dose of 1250 rad. However,

* Present address: Toledo Radiological Associates Inc., Toledo, Ohio 43606.

since the kinetics of development of increased pulmonary nodules after irradiation are markedly different in the rat model used by van den Brenk (van den Brenk *et al.*, 1973; van den Brenk and Kelly, 1974) from the mouse model used by others, it remains a possibility that the lack of a reduction in the effect with fractionated irradiation is a result of a mechanism specific to this rat model. For this reason it was decided to investigate the effect of fractionated irradiation on the incidence of artificial lung metastases after i.v. injection of KHT sarcoma cells in the C3H mouse.

More recently it has been reported that an even greater enhancement of artificial pulmonary metastases can be produced by treatment of the host with cyclophosphamide (CP) before tumour cell injection (van Putten *et al.*, 1975; Carmel and Brown, 1977; Peters and Mason, 1977). It has also been reported that CP can increase spontaneous metastases from a weakly- or non-immunogenic transplanted rat tumour (Moore and Dixon, 1977). Again, however, the doses employed in these studies were single doses and, on the basis of body weight (mg/kg) (although not on surface area), were higher than could be given to humans. It was therefore decided to investigate the effects of fractionation of CP dose on the enhancement of pulmonary nodules in the non-immunogenic KHT/C3H mouse system.

### MATERIALS AND METHODS

In all experiments, female C3H/Km mice, 14–18 weeks old, weighing 28–42 g were used. Ten to 12 mice were preassigned to each treatment group according to weight, so that each group had the same mean weight.

The tumour used was the KHT sarcoma, a tumour that arose spontaneously at the base of the ear of a C3H/Km mouse in 1962, and has since been maintained by serial s.c. passage into syngeneic mice. This tumour in this host is at most only weakly immunogenic, since repeated immunizations with cells sterilized with a dose of 10,000 rad or excision of a growing tumour does not change the number of cells needed to produce tumours in 50% of injected animals (Kallman, Silini and van Putten, 1967), nor affects the number of lung nodules after a given i.v. inoculum of tumour cells (Brown, unpublished). The details of the propagation of the tumours, the preparation of single-cell suspensions and the counting of lung colonies have been described previously (Brown, 1973*b*). Briefly, single-cell suspensions of KHT tumour cells were obtained from s.c. tumours and injected in the recipient mice *via* the tail vein in a volume of 0·2 ml. The animals were killed 17–20 days later and the lungs were extracted and preserved so as to allow the counting of all surface colonies. The mean number of countable colonies was found to be proportional to the number of cells injected in a given experiment, but varied from experiment to experiment for a given number of cells injected (thus necessitating fully self-contained, internally controlled experiments).

For local thoracic irradiation, anaesthetized mice were positioned in a lead box with only the thoracic region exposed. Irradiation conditions were: 250 kVp X-rays; 15 mA; focus skin distance, 44 cm; half value layer, 1·3 mm Cu; and dose rate of ∼100 rad min.

The cyclophosphamide used was Cytoxan™ (Mead Johnson and Co., Evansville, Ind.) and it was dissolved in sterile water immediately before use. Continuous infusion of CP was given *via* the tail vein to non-anaesthetized mice able to eat, drink and move as normal, except that their tails were suspended vertically to an overhead swivelling attachment (Brown and Goffinet, 1970).

### RESULTS

### *Effect of single doses of irradiation to the lungs*

Fig. 1 shows the results of an experiment in which C3H mice irradiated with various doses of X-rays to the thoracic region were injected i.v. with $2 \times 10^4$ KHT tumour cells 48 h after irradiation. The average number of lung colonies per mouse appears to be approximately linearly related to the dose received. A similar result was obtained previously when $5 \times 10^6$ heavily irradiated cells were mixed with the viable tumour cells (Brown, 1973*a*).

In order to determine whether the

FIG. 1.—The number of lung colonies per mouse arising from $2 \times 10^4$ KHT tumour cells injected i.v. 48 h after various doses of thoracic X-irradiation.

FIG. 2.—The number of lung colonies per mouse as a function of the number of KHT tumour cells injected into mice, either sham-irradiated (○) or given 1000 rad (●) to the thorax one day before the tumour cell injection.

FIG. 3.—The number of lung colonies per mouse arising from an i.v. injection of $3 \times 10^4$ KHT tumour cells into mice given single doses of 1000 rad (○) or 2 fractions of 500 rad (●) separated by various intervals. For the groups given 2 fractions of 500 rad, the first dose was given at Time 0 and the second dose at the time shown. The tumour cells were injected at 48 h. The dashed line shows the predicted number of colonies in the fractionated groups based on the 3 single-dose groups if there is no repair of the radiation damage (i.e. no reduction of effect by fractionation).

enhancing effect of prior lung irradiation was constant over a wide range of tumour-cell inocula, mice were given 1000 rad local thoracic irradiation, or sham-irradiated, one day before injection of various numbers of tumour cells. The results are shown on log–log paper (Fig. 2) to illustrate the constant proportionality of increase of lung colonies (by a factor of 8 in this experiment) produced by a dose of 1000 rad. Since the slope of the lines (45°) does not differ significantly from 1 the data are well fitted by straight lines.* Also, since each of these lines extrapolates to the origin on a linear plot (not shown), it is reasonable to conclude that this factor of ~8 in enhancement of lung colonies applies to cell inocula less than those used in this experiment.

### *Effect of divided doses of irradiation*

Fig. 3 shows the results of an experiment in which the effect on the subsequent

* Although the fit to a straight line in the unirradiated lungs is adequate (linearity is not contradicted), the fit is better without the data from the highest cell inoculum ($1 \cdot 6 \times 10^5$ cells). This datum point at the highest inoculum appears to be the result of an effect seen by us (Brown, unpublished) and others (Peters *et al.*, 1978) that the percentage survival of i.v. inoculated cells rises at inoculum sizes greater than $10^5$ cells. This does not affect the data from the irradiated groups, since the highest inoculum used was $4 \times 10^4$ tumour cells.

TABLE I.—*Effect of Dose Fractionation on the Mean Number of Lung Colonies per Mouse (±s.e. mean) following the i.v. Injection of* $2\times10^4$ *KHT Cells*

| Expt No. | No irradiation* | 1000 rad† | 5 × 200 rad‡ | 2000 rad† | 5 × 400 rad‡ |
|---|---|---|---|---|---|
| 1 | 2·05±0·34 | 35·4±3·9 | 38·1±3·3 | — | — |
| 2 | 1·01±0·34 | — | — | 6·6±0·86 | 10·9±3·5 |
| 3 | 1·62±0·31 | — | — | 23·6±1·7 | 25·4±3·2 |

* The "no irradiation" group is the mean of 2 groups given 1 and 5 anaesthetic exposures. No differences between the 2 groups were observed.

† Each mean colony count is the average of 3 groups, injected 42, 48, and 54 h after the single exposure. No significant differences were found between the groups injected at these 3 times.

‡ The fractionated exposures were given at 3h intervals from 42 to 54 h before injection of tumour cells.

development of lung colonies of a thoracic dose of 1000 rad was compared with that of 2 fractions of 500 rad split by intervals varying from 1 to 24 h. In this experiment the first dose of 500 rad was always delivered 48 h before the injection of the tumour cells. Because this meant that the second 500 rad dose was given at varying intervals before the cell injection, it was necessary to have the 1000 rad single-dose points also at varying intervals before cell injection. The broken line of Fig. 3 indicates the expected number of lung colonies in the fractionated groups, based on the single-dose groups obtained at different intervals before cell injection. This predicted line assumes linearity of response between the number of colonies and the irradiation dose. It is apparent from these data that there was little if any reduction in the number of lung colonies produced when a dose of 1000 rad was split into 2 equal fractions of 500 rad.

As a more rigorous test of the effect of dose fractionation on the enhancement of the number of lung colonies by local thoracic irradiation, experiments were performed in which the effects of single doses of either 1000 or 2000 rad were compared with that of 5 equal doses given at 3 h intervals. Table I shows the results obtained. Again, there was no reduction of colony enhancement in the groups which received fractionated irradiation.

*Effect of dose fractionation or infusion of cyclophosphamide*

We have previously reported (Carmel and Brown, 1977) that the dose-response curve for the enhancement of lung nodules in mice given CP one day before cell injection is highly sigmoidal in shape (*i.e.*, very different from the X-ray dose-response curve).

In the experiment to compare the effect of fractionation or continuous infusion of CP with single doses, $2{\cdot}5\times10^3$ KHT tumour cells were injected i.v. either 24, 36, or 48 h after a single injection of CP (200 mg/kg) or 24 h after the completion of a 24 h infusion of 200 mg/kg, or 24 h after the third dose of a course of $3\times66{\cdot}7$ mg/kg of CP at 12 h intervals. The numbers of lung colonies per mouse in these and the control groups are shown in Table II. It can be seen that dividing the dose into 3

TABLE II.—*Effect of Fractionating or Infusing a Cyclophosphamide (CP) Dose of* 200 *mg/kg on the Number of Lung Colonies from an i.v. Injection of* $2{\cdot}5\times10^3$ *KHT Cells*

| Treatment | Time of injection/infusion* (h) | Number of lung colonies/mouse (±s.e. mean) |
|---|---|---|
| Saline injection | 24 | 0·22±0·15 |
| Saline infusion | 0→24 | 0·10±0·10 |
| CP inj. (200 mg/kg) | 0 | 31·2±4·4 |
| CP inj. (200 mg/kg) | 12 | 38·4±4·5 |
| CP inj. (200 mg/kg) | 24 | 30·9±4·3 |
| CP inj. (3 × 66·7 mg/kg) | 0, 12, 24 | 24·9±2·3 |
| CP infusion (200 mg/kg) | 0→24 | 23·3±2·4 |

* The tumour cells were injected at 48 h in all groups.

equal fractions or giving it over a 24 h infusion, slightly reduced the enhancement of lung colonies compared with a single dose of CP. However, this abrogation of the effect was small compared with the remaining marked enhancement over the saline-treated groups.

### DISCUSSION

These experiments show that the enhancement of artificial lung metastases by pulmonary irradiation is not decreased by fractionation of the exposure to fractions as small as 200 rad. This lack of diminution of the effect was also found by van den Brenk and Kelly (1974). In fact, these authors observed a greater response in the fractionated group, although the total dose given was higher than in the single-dose comparison. Thus, if lung colonies arising from a single injection of disaggregated tumour cells are an appropriate model for spontaneous blood-borne metastases, these findings emphasize the clinical relevance of the radiation enhancement of lung colonies, since the doses tested in the present study (1000 and 2000 rad in 5 equal fractions) bracket the doses used in current clinical practice for prophylactic irradiation of the whole lung (Lougheed and Chevalier, 1973; Newton, 1973; Wharam, Phillips and Jacobs, 1974).

The present data also suggest that the mechanism of enhanced pulmonary nodule formation after irradiation is unlikely to be a result of cell killing. This is because, of the possible cell types involved, only lymphocytes are killed by radiation independently of the fractionation pattern, and killed rapidly enough for the effect to become manifest within one day of irradiation (Brown, 1973*a*). However, since the tumour used is non-immunogenic, and the kinetics involved are too rapid for the development of an immune response against the tumour, killing of sensitized lymphocytes can be ruled out. Thus, it would appear that the radiation enhancement of pulmonary nodules is due to a mechanism not involving cell killing, but, to date, no solid evidence for the mechanism is evident.

The fact that fractionation, or extended infusion, of a dose of CP did not produce a large reduction in the number of lung colonies compared with that after the same CP dose in a single injection was unexpected, in view of the marked sigmoid nature of the dose-response curve (Carmel and Brown, 1977). Since the half-life of the cytotoxic effect of CP and its metabolites is very short (15–20 min; Kline *et al.*, 1968), the 3 doses of 67 mg/kg or the 24 h infusion of 200 mg/kg could not have accumulated to produce the same serum level of cytotoxic agents as is produced by a single injection of 200 mg/kg. Rather it is evident that there is a level of "subclinical" damage produced at very low serum concentrations of CP which appears to be approximately linearly related to dose, and which remains susceptible to interaction by further "subclinical" damage to produce the biological response being studied. However, it is clear that this "subclinical" damage does not remain indefinitely: it has been shown that weekly exposures of low doses of CP do not produce a cumulative increase in lung nodules (Carmel and Brown, 1977).

These data on cyclophosphamide, together with the previously published reports of CP inhancement of artificial pulmonary metastases with a variety of tumours (van Putten *et al.*, 1975; Carmel and Brown, 1977; Peters and Mason, 1977), and the recent report of enhanced spontaneous metastasis in rat tumours treated with various doses of CP (Moore and Dixon, 1977), serve to underline the potential hazards of clinical use of CP. Clearly, it is a potent cytotoxic agent and will be beneficial when delivered to pre-existing metastases. However, the hazard of pre-sensitizing the lungs, and possibly other organs, to the subsequent seeding of metastases in them appears to be real. Thus, we feel that in most circumstances it would be wise not to initiate pulmonary irradiation or chemotherapy with cyclophosphamide until after completion of treatment of the primary tumour, whether with surgery or radiotherapy. This would minimize the possibility of viable tumour cells becoming lodged in tissues made extremely receptive to the formation of blood-borne metastases.

We wish to thank Mr E. Parker for his excellent technical assistance, and Dr L. J. Peters, who suggested the fractionation and infusion experiments with CP. These investigations were supported by Public Health Service Research Grants CA-15201 and CA-10372 from the National Cancer Institute, Department of Health, Education and Welfare.

### REFERENCES

Brown, J. M. (1973*a*) The Effect of Lung Irradiation on the Incidence of Pulmonary Metastases in Mice. *Br. J. Radiol.*, **46**, 613.

Brown, J. M. (1973*b*) A Study of the Mechanism by which Anticoagulation with Warfarin Inhibits Blood-borne Metastases. *Cancer Res.*, **33**, 1217.

Brown, J. M. & Goffinet, D. (1970) A Technique for Intra-arterial Infusion of Tumor-bearing Mice. *J. Lab. clin. Med.*, **76**, 175.

Carmel, R. J. & Brown, J. M. (1977) The Effect of Cyclophosphamide and Other Drugs on the Incidence of Pulmonary Metastases in Mice. *Cancer Res.*, **37**, 145.

Fidler, I. J. & Zeidman, I. (1972) Enhancement of Experimental Metastasis by X-ray: a Possible Mechanism. *J. Med.* (*Basel*), **3**, 172.

Kallman, R. F., Silini, G. & van Putten, L. M. (1967) Factors Influencing the Quantitative Estimation of the *In vivo* Survival of Cells from Solid Tumors. *J. natn Cancer Inst.*, **39**, 539.

Kline, I., Gang, M., Tyrer, D. D., Mantel, N., Venditti, J. M. & Goldin, A. (1968) Duration of Drug Levels in Mice as Indicated by Residual Antileukemic Efficiency. *Chemotherapy*, **13**, 28.

Lougheed, M. N. & Chevalier, L. (1973) Prophylactic Radiotherapy to Prevent Pulmonary Metastases. Abstract 810, *Proc. 8th Int. Congr. Radiol.*, Madrid. Amsterdam: Excerpta Medica.

Moore, J. V. & Dixon, B. (1977) Metastasis of a Transplantable Mammary Tumour in Rats Treated with Cyclophosphamide and/or Irradiation. *Br. J. Cancer*, **36**, 221.

Newton, K. A. (1973) Prophylactic Irradiation of the Lung in Bone and Soft Tissue Sarcoma. The Colton Papers No. XXIV. In *Bone—Certain Aspects of Neoplasia*. Ed. C. H. G. Price and F. G. M. Ross. London: Butterworth. p. 307.

Owen, L. N. & Bostock, D. E. (1973) Prophylactic X-irradiation of the Lung in Canine Tumours with Particular Reference to Osteosarcoma. *Eur. J. Cancer*, **9**, 747.

Peters, L. J. (1974) The Potentiating Effect of Prior Local Irradiation of the Lungs on the Development of Pulmonary Metastases. *Br. J. Radiol.*, **47**, 827.

Peters, L. J. & Mason, K. (1977) Enhancement of Artificial Lung Metastases by Cyclophosphamide: Pharmacological and Mechanistic Considerations. In *Cancer Invasion and Metastasis: Biologic Mechanisms and Therapy*. Ed. S. B. Day *et al.* New York: Raven Press. p. 397.

Peters, L. J., Mason, K., McBride, W. H. & Patt, Y. Z. (1978) Enhancement of Lung Colony-forming Efficiency by Local Thoracic Irradiation: Interpretation of Labeled Cell Studies. *Radiology*, **126**, 499.

Thompson, S. C. (1974) Tumour Colony Growth in the Irradiated Mouse Lung. *Br. J. Cancer*, **30**, 337.

van den Brenk, H. A. S., Burch, W. M., Orton, C. & Sharpington, C. (1973) Stimulation of Clonogenic Growth of Tumour Cells and Metastases in the Lungs by Local X-radiation. *Br. J. Cancer*, **27**, 291.

van den Brenk, H. A. S. & Kelly, H. (1974) Potentiating Effect of Prior Local Irradiation of the Lungs on Pulmonary Metastases. *Br. J. Radiol.*, **47**, 332.

van Putten, L. M., Kram, L. K. J., van Dierendonck, H. H. C., Smink, T. & Fuzy, M. (1975) Enhancement by Drugs of Metastatic Lung Nodule Formation after Intravenous Tumour Cell Injection. *Int. J. Cancer*, **15**, 588.

Wharam, M. D., Phillips, T. L. & Jacobs, E. M. (1974) Combination Chemotherapy and Whole Lung Irradiation for Pulmonary Metastases from Sarcomas and Germinal Cell Tumors of the Testis. *Cancer*, **34**, 136.

Withers, H. R. & Milas, L. (1973) Influence of Preirradiation of Lung on Development of Artificial Pulmonary Metastases of Fibrosarcoma in Mice. *Cancer Res.*, **33**, 1931.